# Usefulness of Immunocytochemical Staining for Brachyury in Cytodiagnosis of Conventional Chordoma

**DOI:** 10.3390/diagnostics16131985

**Published:** 2026-06-25

**Authors:** Naoto Kohno, Mitsuaki Ishida, Shizuka Ono, Mayumi Uragami, Chihiro Deguchi, Reika Takeda, Yoshinobu Hirose

**Affiliations:** 1 Department of Pathology, Osaka Medical and Pharmaceutical University, Takatsuki City 569-8686, Osaka, Japan; naoto.kono@ompu.ac.jp (N.K.); shizuka.akashi@ompu.ac.jp (S.O.); yoshinobu.hirose@ompu.ac.jp (Y.H.); 2 Division of Pathology, Osaka Medical and Pharmaceutical University Hospital, Takatsuki City 569-8686, Osaka, Japan; mayumi.uragami@ompu.ac.jp (M.U.); chihiro.deguchi@ompu.ac.jp (C.D.); reika.takeda@ompu.ac.jp (R.T.)

**Keywords:** conventional chordoma, cytology, immunocytochemical staining, brachyury

## Abstract

**Background/Objectives**: Conventional chordomas are rare malignant bone tumors characterized by brachyury expression. The presence of polygonal-to-epithelioid neoplastic cells containing rich cytoplasm and relatively large nuclei in a myxoid background is a characteristic cytological feature of conventional chordomas. Physaliphorous cells, characterized by the presence of multiple well-marginated vacuoles within the cytoplasm, are also characteristic of this rare bone tumor. However, the cytological diagnosis of conventional chordomas can be challenging because of their rarity and lack of specific features. Therefore, useful immunocytochemical markers are required. Immunohistochemical staining for brachyury has been widely used for diagnosis; however, immunocytochemical staining using cytological specimens has not yet been performed. The purpose of this study was to evaluate the usefulness of the immunocytochemical staining for brachyury in cytodiagnosis of conventional chordoma. **Methods**: This study included patients diagnosed with conventional chordoma based on postoperative histopathological findings who underwent intraoperative squash cytological examination. Cytological features and immunocytochemical staining for brachyury were evaluated. **Results**: Two patients with conventional chordoma were included. The tumor extended from the lumbar spine to the cauda equina and clivus. Cytological examination revealed the presence of small sheets and isolated polygonal neoplastic cells with rich cytoplasm and round nuclei with inconspicuous nucleoli in a myxoid background. Cellular clusters were noted in one specimen and physaliphorous cells were observed in the other. Immunocytochemical staining for brachyury revealed positive nuclear expression in both specimens. **Conclusions**: Immunocytochemical staining for brachyury could provide useful information for the cytodiagnosis of this rare tumor, even in cases lacking typical cytological features.

## 1. Introduction

Conventional chordomas are rare malignant bone tumors that show a phenotype recapitulating that of the notochord [1]. This tumor occurs in less than one case per million person-years in the axial skeleton, including the coccyx, sacrum, and skull base [1]. The characteristic histopathological features of this tumor are the lobular proliferation of large epithelioid neoplastic cells, arranged as cords and nests, and embedded in a rich extracellular myxoid matrix [1]. These neoplastic cells have clear-to-light eosinophilic cytoplasm and may exhibit characteristic bubbly cytoplasm, known as physaliphorous cells [1]. The hallmark of conventional chordoma is brachyury expression, which is encoded by the *T-Box transcription factor T* (*TBXT*), a transcription factor required for notochordal development [1,2,3,4,5]. Therefore, immunohistochemical demonstration of brachyury is a useful diagnostic marker for conventional chordoma [2,3,4].

Cytological examination is performed to diagnose conventional chordoma. Several studies have reported the cytological features of conventional chordoma [6,7,8,9,10,11,12,13,14,15,16,17]. Most of these studies included single cases or fewer than 10 cases [6,7,8,9,10,11,12,13,14,15,16]; however, the largest cytological report, containing 47 lesions from 44 patients, has recently been published [17]. The cytological features of conventional chordoma are as follows: (i) cellular smears composed of polygonal to epithelioid cells in a rich myxoid background or chondromyxoid material, showing metachromasia; (ii) these cells appear as isolated single cells, vague trabecular cords or syncytial clusters, and have relatively large nuclei with finely granular chromatin and inconspicuous to variably large nucleoli; (iii) neoplastic cells have rich cytoplasm and may have multiple well-marginated vacuoles known as physaliphorous cells; and (iv) necrosis and mitosis are uncommon [6,7,8,9,10,11,12,13,14,15,16,17]. However, some cytological reports have shown that conventional chordomas occasionally lack physaliphorous cells [10,14]. Additionally, a myxoid or chondromyxoid background and intracytoplasmic vacuoles are not specific to conventional chordomas [17]. Accordingly, the cytological diagnosis of conventional chordomas may occasionally be difficult [9,17]; therefore, a useful immunocytochemical marker is required. Previous studies have reported immunohistochemical staining for brachyury using cell-block materials (formalin-fixed and paraffin-embedded materials obtained during cytological examination) [6,17,18]; however, the usefulness of immunocytochemical staining for this marker using cytological specimens has not been analyzed. In this study, we examined the usefulness of immunocytochemical staining for brachyury using cytological specimens for the diagnosis of conventional chordoma.

## 2. Materials and Methods

### 2.1. Patient Selection

This study included patients diagnosed with conventional chordoma through postoperative pathological examination at Osaka Medical and Pharmaceutical University Hospital (Osaka, Japan) who underwent squash cytological examination at the time of intraoperative examination between January 2020 and December 2024. The inclusion criteria were as follows: histopathological confirmation of conventional chordoma, performance of intraoperative examination, and availability of specimens for immunocytochemical staining for brachyury. The following patients were excluded from this study: patients without sufficient cytological specimens for examination of cytological features and immunocytochemical staining.

This retrospective single-institution study was conducted according to the Declaration of Helsinki guidelines. The study protocol was approved by the Institutional Review Board of Osaka Medical and Pharmaceutical University Hospital (approval no. 2023-073, approval date: 1 April 2026). The Institutional Review Board waived the requirement for informed consent owing to the retrospective study design; the medical records and archived samples were used without risk to the participants. All data were anonymized. Moreover, this study excluded children. Information regarding the inclusion criteria and opportunity to opt out was provided on the institutional website (https://www.ompu.ac.jp/u-deps/path/img/file50.pdf, accessed on 22 April 2026).

### 2.2. Cytological Analysis

Squash cytological specimens were stained with Papanicolaou and Giemsa stains. We evaluated the cytological characteristics of the specimens, such as background features (presence of myxoid material) and neoplastic cell features.

### 2.3. Histopathological Analysis

Surgically resected bone tumor specimens were fixed in 10% buffered neutral formalin, dehydrated, paraffin-embedded, sectioned, and stained with hematoxylin and eosin. At least two researchers independently evaluated the histopathological features of all specimens, including the epithelial type and the presence of myxoid material, and then compared them with the cytological features of the squash specimens.

### 2.4. Immunohistochemical and Immunocytochemical Analyses

We performed immunohistochemical analysis using an autostainer (Discovery Ultra System; Roche Diagnostics, Basel, Switzerland), and the Optivew DAB Universal Kit (cat. no. 518-111427; Roche Diagnostics) was used for immunostaining. A rabbit monoclonal antibody against brachyury (EPR18113; Abcam, Cambridge, UK; diluted 1:200) was used.

Immunocytochemical analysis was performed using the same method, and a primary antibody was used for immunohistochemical analysis. In this study, Papanicolaou-stained cytological specimens were reused for immunocytochemical staining. Papanicolaou-stained cytological specimens were decolorized (50% ethanol containing 0.5% hydrochloric acid for 1 day at room temperature) and then reused for immunocytochemical staining. Inflammatory cells present in the cytological specimens were used as an internal negative control for immunocytochemical staining for brachyury. Nuclear expression was considered positive immunocytochemical staining for brachyury.

## 3. Results

### 3.1. Patient Characteristics

A total of three patients were identified during the study period, and one patient was excluded because of inadequate cytological specimens. Two patients were included in this study, and their clinicocytological features are summarized in Table 1.

Patient 1. A 51-year-old Japanese female presented with headache and fatigue. Computed tomography revealed the presence of a tumorous lesion from the lumbar spine to the cauda equina. The tumor was surgically resected.

Patient 2. A 63-year-old Japanese female presented with amblyopia when looking to the right. Computed tomography revealed a tumor in the clivus. Subsequently, surgical resection was performed.

### 3.2. Cytological Features

**Figure 1:**
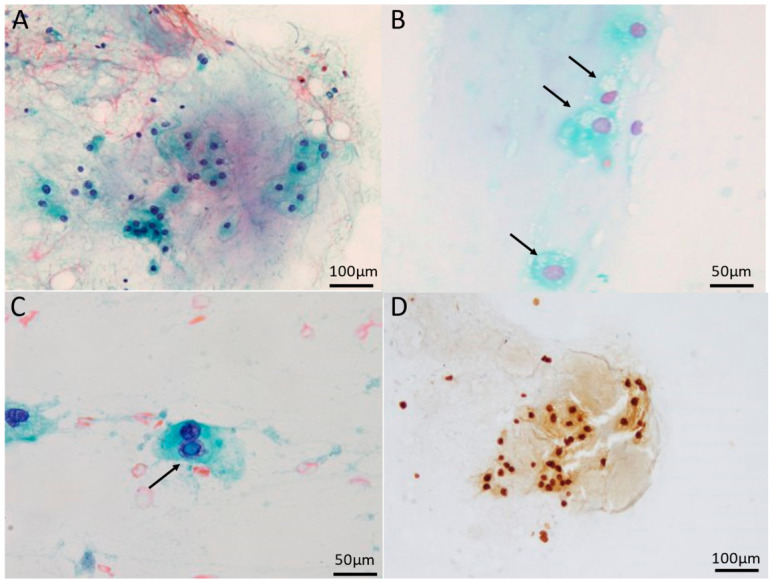
Cytological and immunocytochemical features of Patient 1. (**A**) Small clusters and isolated neoplastic cells are present in a background of myxoid material. These cells have rich granular cytoplasm and round nuclei (Papanicolaou staining, ×200). (**B**) Large neoplastic cells with multiple marginated vacuoles (physaliphorous cells) are observed (arrows) (Papanicolaou stain, ×400). (**C**) A binucleated cell with an intranuclear inclusion (arrow) is observed. (Papanicolaou staining, ×400) (**D**) Neoplastic cells show positive nuclear expression for brachyury (×200).

**Figure 2:**
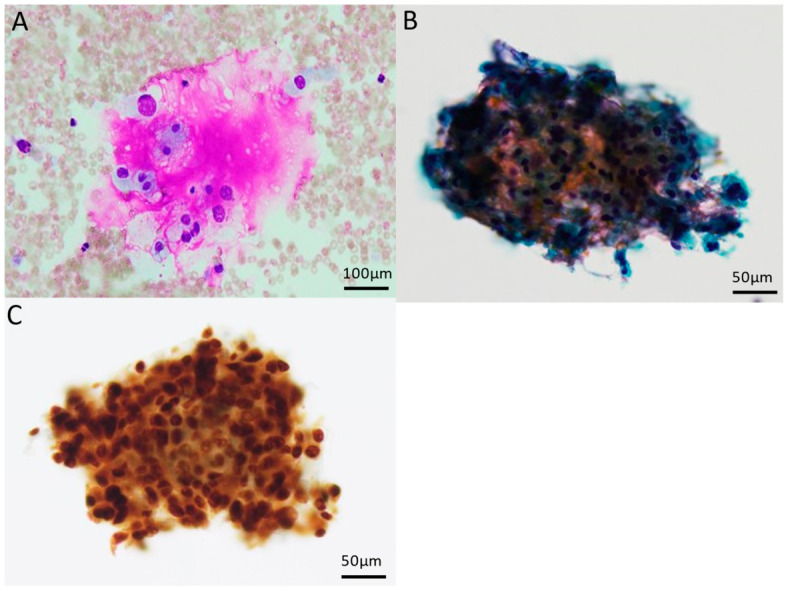
Cytological and immunocytochemical features of Patient 2. (**A**) Small clusters and isolated neoplastic cells are present in a background of myxoid material that shows metachromasia (Giemsa stain, ×200). (**B**) Presence of a tightly aggregated cluster of neoplastic cells (Papanicolaou staining, ×400). (**C**) Immunocytochemical staining demonstrates that neoplastic cells show positive nuclear expression for brachyury (×400).

Table 1 summarizes the cytological features of both patients. The specimens from both patients contained sufficient amounts of well-preserved cellular components. Small clusters and isolated neoplastic cells appeared in a myxoid background in both specimens (Figure 1A,B and Figure 2A). Tightly aggregated cellular clusters were present in the specimen from Patient 2 (Figure 2B), but not in that from Patient 1. The myxoid material exhibited metachromasia on Giemsa staining (Figure 2A).

Necrosis was not observed in either specimen. These neoplastic cells were polygonal, with clear cytoplasmic borders, a relatively rich cytoplasm, and round nuclei containing inconspicuous nucleoli (Figure 1A,B and Figure 2A). Large neoplastic cells with marginated vacuoles, consistent with physaliphorous cells, were observed in the specimen from Patient 1 (Figure 1B) but not in the specimen from Patient 2. Binucleated cells were observed in both specimens (Figure 1C); however, multinucleated cells were absent. Intranuclear inclusions were observed in both specimens (Figure 1C). No mitotic figures and high-grade sarcomatous components were observed in either specimen.

### 3.3. Immunocytochemical Features

Brachyury was diffusely expressed in the nuclei of neoplastic cells in both lesions (Figure 1D and Figure 2C). Accordingly, these two lesions were cytologically diagnosed as conventional chordomas.

### 3.4. Histopathological and Immunohistochemical Features

**Figure 3:**
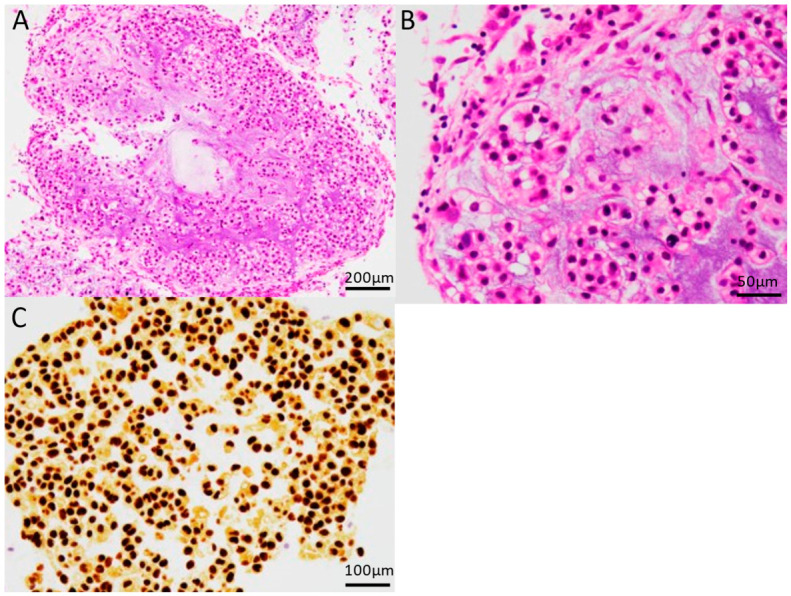
Histopathological features of the resected specimen from Patient 1. (**A**) Trabecular or nested proliferation of polygonal cells in a myxoid background (hematoxylin and eosin, ×100). (**B**) These neoplastic cells have rich eosinophilic-to-clear cytoplasm and round nuclei containing inconspicuous nucleoli (hematoxylin and eosin, ×400). (**C**) Diffuse positive nuclear immunoreactivity for brachyury in the neoplastic cells (×200).

The resected bone tumors from both patients showed fundamentally similar histopathological characteristics. Proliferation of polygonal neoplastic cells forming cords or small nests within a myxoid-rich material was observed (Figure 3A). These neoplastic cells had round nuclei without conspicuous nucleoli and rich granular eosinophilic-to-clear cytoplasm (Figure 3B), including large neoplastic cells with marginal vacuoles (physaliphorous cells) (Figure 3B). However, mitotic figures and necrosis were not observed.

Regarding the correlation with the cytological features, the presence of the polygonal neoplastic cells having rich cytoplasm within the myxoid-rich material reflected the cytological features of both patients. Although both tumors had physaliphorous cells in the histopathological specimens, the cytological specimens from Patient 2 lacked these characteristic cells.

Immunohistochemical analysis revealed that the neoplastic cells in both tumors showed diffuse nuclear positive immunoreactivity for brachyury (Figure 3C).

Accordingly, a diagnosis of conventional chordoma was made in both tumors.

## 4. Discussion

In this study, we demonstrated, for the first time, the usefulness of positive immunocytochemical staining for brachyury in conventional chordomas using cytological specimens. The cytological features of conventional chordomas have been recognized; however, the characteristic features, including myxoid material and polygonal neoplastic cells with intracytoplasmic vacuoles, are not specific. Moreover, these findings are not observed in all cases, as demonstrated by Patient 2 in the present study. Therefore, the cytological diagnosis of conventional chordomas may be difficult in some cases because of the aforementioned factors and their rarity [10,14,17]. Our findings revealed that immunocytochemical staining for brachyury could provide useful information for the diagnosis of conventional chordomas, even in cases lacking typical cytological features.

Brachyury (‘short tail’ in Greek), a transcription factor encoded by the *TBXT* on chromosome 6q27 [5], plays a key role in embryogenesis, particularly in the posterior development of the mesoderm and cellular differentiation of chordates [19]. Brachyury expression is a hallmark of chordomas [1,2,3,4,5], and most adult human tissues lack its expression [20]. Chordomas are classified as conventional, dedifferentiated (containing both conventional chordomas and high-grade sarcomas), and poorly differentiated (characterized by poorly differentiated neoplasms with notochordal differentiation) [1,21,22]. Brachyury is expressed in conventional chordoma, as well as the conventional chordoma component of dedifferentiated chordoma and poorly differentiated chordoma [1,21,22]. Miettinen et al. analyzed brachyury expression in 5229 cases with various tumor types [23]. Brachyury expression was absent in most carcinomas and sarcomas but was present in 41% (12/29 cases) of small cell neuroendocrine carcinomas of the lung. Moreover, embryonal carcinomas (74%, 25/34 cases), seminomas (46%, 33/71 cases), and yolk sac tumors (16%, 1/6 cases) of the testes were positive for brachyury immunoreactivity [23]. A recent immunohistochemical study, in which brachyury expression was analyzed in 14,976 malignant tumors across 135 tumor entities and 608 samples from 76 normal tissues, reported findings consistent with these results [20]. According to the results, brachyury expression was not detected in any of the 76 analyzed normal tissues, including skeletal muscle, heart, lung, gastrointestinal tract, liver, pancreas, ovary, uterus, kidney, prostate, and testis [20]. In the neoplastic lesions, only 6 of 135 tumor entities showed positive immunoreactivity for brachyury [20]. All conventional chordomas (10/10 cases), 21.4% (9/42 cases) of yolk sac tumors of the testis, 15.2% (7/46 cases) of embryonal carcinoma of the testis, 4.4% (25/562 cases) of seminoma of the testis, 2.4% (1/41 cases) of teratoma of the testis, and 0.1% (2/2280 cases) of adenocarcinoma of the colon showed brachyury expression [20]. Moreover, a small proportion of non-small cell lung and digestive system carcinomas have also been shown to express brachyury (4%, 2%, and 3% of adenocarcinomas of the lung, stomach, and pancreas, respectively) [23,24]. In contrast, brachyury expression is absent in chondrosarcomas and myoepithelial tumors (0/54, 0/18, and 0/11 cases of chondrosarcoma of the bone, extraskeletal myxoid chondrosarcoma, and myoepithelial tumors of the soft tissue, respectively) [23,25], which are the main differential diagnostic considerations for conventional chordomas. Accordingly, brachyury expression is not a highly specific marker for conventional chordomas; however, it is useful in their diagnosis.

Immunohistochemical staining using cell block materials has been well recognized as a useful method for cytological diagnosis; however, not all cytological materials are prepared for this method. In contrast, immunocytochemical staining can be performed using pre-existing cytological slides for cytological diagnosis, as shown in the present study. Thus, previous reports have demonstrated the usefulness of immunocytochemical staining for some types of tumors, especially those characterized by the presence of a specific marker, such as brachyury, for conventional chordoma. For example, solitary fibrous tumor (SFT) is a relatively rare mesenchymal tumor, characterized by the proliferation of spindle neoplastic cells with branching, dilated, and thin-walled vessels [26]. SFT and hemangiopericytoma were previously considered to be distinct tumor entities; however, it has been shown that most cases exhibit the characteristic NGFI-A-binding protein 2 (*NAB2*)-signal transducer and activator of transcription 6 (*STAT6*) gene fusion [27,28]. Accordingly, these two entities have been recognized as a single disease entity, namely, SFT [26]. SFT shows a wide histopathological spectrum, ranging from a hypocellular type with predominant collagenous stroma to a highly cellular type with less fibrous stroma [26,29]. The former type typically occurs in the pleura, and the latter usually develops at the extra-pleural sites, such as the dura mater [26,29]. Most cases of both types of SFT have the *NAB2–STAT6* fusion [26,27,28,29]. The immunohistochemical hallmark of SFT is positive immunoreactivity for STAT6, and this marker is considered to be specific for SFT [26,29]. Some studies have demonstrated the usefulness of immunocytochemical staining for STAT6 in the cytodiagnosis of SFT, because this type of tumor shows non-specific cytological features, and its differential cytological diagnosis from other spindle tumors is required [30,31]. Another example of the usefulness of immunocytochemical staining is pan-tyrosine receptor kinase (Trk) for secretory carcinoma of the salivary gland [32]. Secretory carcinoma of the salivary gland was originally termed mammary analog secretory carcinoma, and is characterized by the presence of the ETS variant transcription 6 (*ETV6*)-Neurotrophic receptor tyrosine kinase 3 (*NTRK3*) gene fusion, with a small subset of cases with *ETV6*—Rearranged during transfection (*RET*) or *ETV6*—Mastermind-like 3 (*MAML3*) gene fusions [33]. Approximately 90% of secretory carcinomas harbor a characteristic chromosomal rearrangement, t(12;15)(p13;q25), resulting in the *ETV6-NTRK3* fusion [33]. The diagnosis of secretory carcinoma has traditionally been determined by detecting the aforementioned fusion genes using fluorescence in situ hybridization or DNA sequencing; however, it has been clearly demonstrated that immunohistochemical analysis for pan-Trk, which can detect the abnormal fusion protein produced by the *NTRK* fusion gene, is useful for detecting *NTRK* gene fusion [34]. It has been demonstrated that 20 of 21 cases of secretory carcinoma of the salivary gland with NTRK gene fusions showed positive nuclear reactivity for pan-Trk by immunohistochemistry [34]. Thus, this marker is recognized as useful for the histopathological diagnosis of secretory carcinoma, allowing its distinction from histopathological mimics, including acinic cell carcinoma of the salivary gland. Immunocytochemical staining for pan-Trk in the cytological diagnosis of secretory carcinoma of the salivary gland has been described [32]. In that study, all eight secretory carcinomas showed positive immunoreactivity for pan-Trk and negative immunoreactivity for nuclear receptor subfamily 4 group A member 3 (NR4A3), a specific marker for acinic cell carcinoma of the salivary gland, using cytological slides [32]. Although not all secretory carcinomas (approximately 90%) have *NTRK* gene fusion, immunocytochemical staining for pan-Trk using cytological slides is a useful method for the cytological diagnosis of secretory carcinoma of the salivary gland [32]. Moreover, TRK inhibitors have been reported to show good responses in tumors harboring *NTRK* gene fusions [35]; therefore, immunocytochemical analysis of pan-Trk might be useful for the selection of patients for treatment with this inhibitor. Accordingly, immunocytochemical staining is a useful method for cytological diagnosis, especially for neoplasms that have a specific marker. The present study demonstrated the usefulness of immunocytochemical staining for brachyury in the cytological diagnosis of conventional chordoma; this method can be applied to other lesions with specific diagnostic markers for cytological diagnosis, as well as to guide therapeutic strategies.

Previous studies have shown that immunohistochemical staining for brachyury using cell block materials is useful for the cytodiagnosis of conventional chordoma [6,17,18]. Immunocytochemical staining using cytological slides of conventional chordoma has been reported for cytokeratin, vimentin, epithelial membrane antigen, and glial fibrillary acidic protein [15], as well as carcinoembryonic antigen, S-100 protein, and neuron-specific enolase [16]. However, no study has reported the usefulness of immunocytochemical staining for brachyury using cytological slides. To the best of our knowledge, the present study is the first to demonstrate brachyury expression in neoplastic cells through immunocytochemical staining using cytological slides.

The characteristic cytological features of conventional chordomas have been recognized [6,7,8,9,10,11,12,13,14,15,16,17]; therefore, the cytological diagnosis of this type of tumor has been reported to have high accuracy in a large case series [17]. Wakely et al. analyzed 47 cytological cases (43 fine-needle aspiration specimens and four imprint smears), and 91% (43 of 47 cases) were accurately diagnosed as conventional chordoma [17]. Although no detailed data regarding the frequency of physaliphorous cells and a rich myxoid background or chondromyxoid material showing metachromasia were available, cytological diagnosis is one of the useful methods for the diagnosis of conventional chordoma. However, uncommon cytological findings, such as the absence of physaliphorous cells and the presence of pleomorphic cells, have been reported in some cases [6,7,9,10,14]. Rekhi and Karmarkar reported nine cases describing the cytological features of conventional chordoma and showed that myxoid stroma was present in all cases, physaliphorous cells were present in eight of nine cases, and pleomorphic cells were present in two of the nine cases [9]. Singh and Rao reported the cytological features of squash smears of 12 cases of conventional chordoma and demonstrated that 2 of 12 cases contained no physaliphorous cells in the cytological specimens [7]. Moreover, neoplastic cells with intracytoplasmic vacuoles have been observed in conventional chordomas, except for physaliphorous cells that resemble signet ring cells [8,12]. Therefore, the cytological differential diagnoses include conventional chondrosarcoma, myoepithelioma, and metastatic carcinoma [9,17,18]. Conventional chondrosarcomas, myoepitheliomas, and conventional chordomas have a rich myxoid or chondromyxoid material in the background; nevertheless, the presence of physaliphorous cells can help differentiate chordomas from conventional chondrosarcomas and myoepitheliomas [9,17]. Cellular clusters, as seen in the present specimen, are sometimes observed, and signet ring cells can be present in cytological specimens of conventional chordomas [8,9,12]. Therefore, differentiation from metastatic carcinoma, especially adenocarcinoma and mucinous adenocarcinoma, is sometimes required [9]. The presence of physaliphorous cells can help differentiate conventional chordomas from these metastatic carcinomas [9]. Moreover, immunostaining for cytokeratin and S-100 protein may not be useful for the differential diagnosis because S-100 protein is expressed in myoepithelioma, conventional chordomas, and conventional chondrosarcoma, and cytokeratin expression is noted in conventional chordomas, myoepitheliomas, and metastatic carcinomas [9,18]. Therefore, immunocytochemical staining for brachyury may be useful for the cytological diagnosis of conventional chordoma and for differentiating it from these diagnostic considerations, because brachyury is not expressed in conventional chondrosarcoma, myoepithelioma, and the vast majority of carcinomas, as mentioned above [23,24,25]. Accordingly, immunocytochemical staining for this marker using cytological specimens, together with consideration of the cytological features, may facilitate the rapid and correct diagnosis of conventional chordoma. However, small cell neuroendocrine carcinomas, embryonal carcinomas, seminomas, yolk sac tumors, and a small proportion of adenocarcinomas can show positive expression for brachyury, which leads to a pitfall of immunocytochemical staining for this marker; thus, consideration of the cytological features is important for an accurate diagnosis.

This study has some limitations. First, this study included only two cytological cases of conventional chordoma. As this was a pilot study, the results cannot be generalized to the usefulness of immunohistochemical staining for brachyury. Further investigation with a larger, multi-institutional cohort is required to validate its usefulness, particularly given the rarity of this tumor (less than one case per one million person-years). Second, immunocytochemical analysis for brachyury was not performed to address differential diagnostic considerations of conventional chordoma, such as conventional chondrosarcoma and myoepithelial tumors, because of the unavailability of squash cytological specimens from these rare tumors. However, as previously mentioned, absence of brachyury expression has been reported in these tumors [23,25]; thus, immunocytochemical staining for brachyury, together with consideration of the cytological features, may be useful for the cytological diagnosis of conventional chordoma. Moreover, brachyury expression has been noted in some types of malignant tumors, including small cell carcinoma of the lung and testicular tumors; however, careful evaluation of cytological features may help avoid potential false–positive interpretations of brachyury.

## 5. Conclusions

Only two cytological specimens were available in this study; however, the findings showed that immunocytochemical staining for brachyury could provide useful information for the cytological diagnosis of conventional chordomas. Brachyury is not a highly specific marker for conventional chondromas; however, it is expressed in most conventional chondromas. Other types of tumors expressing brachyury are limited. Therefore, a combination of cytological features and immunocytochemical staining for brachyury can lead to the correct diagnosis of conventional chordomas.

## Figures and Tables

**Table 1:** Clinicocytological features of conventional chordoma in the present study.

	Age	Sex	Location	Cytological Features				Immunocytochemistry for Brachyury
				Background	Cluster of Neoplastic Cells	Shape of the Neoplastic Cells	Physaliphorous Cells	
Patient 1	51	Female	Lumber spine to cauda equina	Myxoid	Small clusters and isolated cells	Polygonal neoplastic cells having rich cytoplasm and round nuclei	+	+
Patient 2	63	Female	Clivus	Myxoid	Aggregated cellular clusters and small clusters	Polygonal neoplastic cells containing rich cytoplasm and round nuclei	−	+

## Data Availability

The original contributions presented in this study are included in the article. Further inquiries can be directed to the corresponding author.

## Abbreviations

The following abbreviations are used in this manuscript:

ETV6	ETS variant transcription 6
MAML3	Mastermind-like 3
NAB2	NGFI-A-binding protein 2
NR4A3	Nuclear receptor subfamily 4 group A member 3
NTRK3	Neurotrophic receptor tyrosine kinase 3
RET	Rearranged during transfection
SFT	solitary fibrous tumor
STAT6	Signal transducer and activator of transcription 6
TBXT	T-box transcription factor T
Trk	tyrosine receptor kinase
